# 3-(1*H*-1,3-Benzimidazol-2-yl)-2,7-dimeth­oxy­quinoline

**DOI:** 10.1107/S1600536812032357

**Published:** 2012-07-18

**Authors:** Hayette Alliouche, Sofiane Bouacida, Thierry Roisnel, Ali Belfaitah

**Affiliations:** a Laboratoire des Produits Naturels d’Origine Végétale et de Synthèse Organique (PHYSYNOR), Université Mentouri-Constantine, 25000 Constantine, Algeria; b Unité de Recherche de Chimie de l’Environnement et Moléculaire Structurale (CHEMS), Université Mentouri-Constantine, 25000 Algeria; c Département Sciences de la Matière, Faculté des Sciences Exactes et Sciences de la Nature et de la Vie, Université Oum El Bouaghi, Algeria; d Centre de Difractométrie X, UMR 6226 CNRS Unité Sciences Chimiques de Rennes, Université de Rennes I, 263 Avenue du Général Leclerc, 35042 Rennes, France

## Abstract

In the title mol­ecule, C_18_H_15_N_3_O_2_, the dihedral angle between the quinoline and benzimidazole ring systems is 23.57 (5)°. The C atoms of the meth­oxy groups are both close to being coplanar with their attached ring systems [deviations = 0.193 (2) and −0.020 (2) Å]. An intra­molecular N—H⋯O hydrogen bond closes an *S*(6) ring. In the crystal, N—H⋯N hydrogen bonds link the mol­ecules into *C*(4) chains propagating in [010]. Weak C—H⋯π inter­actions also occur.

## Related literature

For our previous work on the preparation of functionalized heterocyclic compounds with potential biological activity, see: Benzerka *et al.* (2012 ▶); Hayour *et al.* (2011 ▶). For further synthetic details, see: Fioraventi *et al.* (2006 ▶).
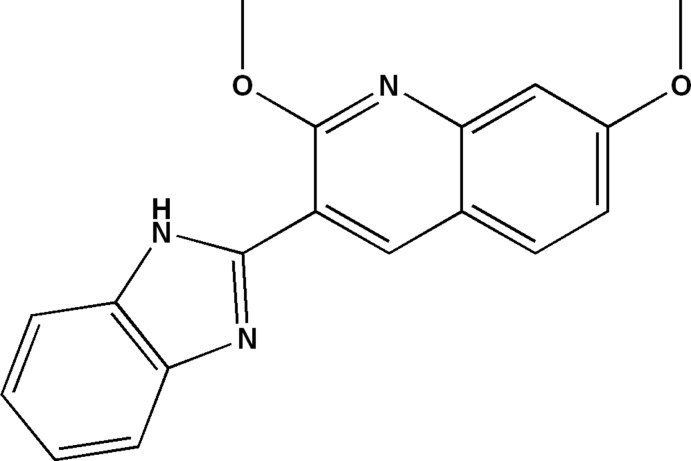


## Experimental

### 

#### Crystal data

C_18_H_15_N_3_O_2_
*M* *_r_* = 305.33
Orthorhombic, 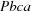

*a* = 6.7094 (2) Å
*b* = 9.4134 (3) Å
*c* = 49.1620 (16) Å
*V* = 3104.99 (17) Å^3^
*Z* = 8
Mo *K*α radiation
μ = 0.09 mm^−1^
*T* = 150 K
0.51 × 0.29 × 0.09 mm

#### Data collection

Bruker APEXII diffractometer
Absorption correction: multi-scan (*SADABS*; Sheldrick, 2002 ▶) *T*_min_ = 0.900, *T*_max_ = 0.992
14322 measured reflections
3398 independent reflections
2696 reflections with *I* > 2σ(*I*)
*R*_int_ = 0.035

#### Refinement

*R*[*F* ^2^ > 2σ(*F* ^2^)] = 0.046
*wR*(*F* ^2^) = 0.112
*S* = 1.03
3398 reflections
210 parameters
H-atom parameters constrained
Δρ_max_ = 0.23 e Å^−3^
Δρ_min_ = −0.21 e Å^−3^

### 

Data collection: *APEX2* (Bruker, 2001 ▶); cell refinement: *SAINT* (Bruker, 2001 ▶); data reduction: *SAINT*; program(s) used to solve structure: *SIR2002* (Burla *et al.*, 2005 ▶); program(s) used to refine structure: *SHELXL97* (Sheldrick, 2008 ▶); molecular graphics: *ORTEP-3* (Farrugia, 1997 ▶) and *DIAMOND* (Brandenburg & Berndt, 2001 ▶); software used to prepare material for publication: *WinGX* (Farrugia, 1999 ▶).

## Supplementary Material

Crystal structure: contains datablock(s) global, I. DOI: 10.1107/S1600536812032357/hb6897sup1.cif

Structure factors: contains datablock(s) I. DOI: 10.1107/S1600536812032357/hb6897Isup2.hkl

Supplementary material file. DOI: 10.1107/S1600536812032357/hb6897Isup3.cml

Additional supplementary materials:  crystallographic information; 3D view; checkCIF report

## Figures and Tables

**Table 1:** Hydrogen-bond geometry (Å, °) *Cg*1, *Cg*2 and *Cg*4 are the centroids of the N16/N17/C15/C18/C23, N4/C3/C5/C12–C14 and C18–C23 rings, respectively.

*D*—H⋯*A*	*D*—H	H⋯*A*	*D*⋯*A*	*D*—H⋯*A*
N16—H16⋯N17^i^	0.88	2.02	2.8397 (17)	154
N16—H16⋯O2	0.88	2.27	2.7107 (17)	111
C1—H1*A*⋯*Cg*2^ii^	0.98	2.67	3.3101 (18)	123
C1—H1*C*⋯*Cg*1^iii^	0.98	2.82	3.4955 (17)	127
C20—H20⋯*Cg*4^iv^	0.95	2.99	3.8271 (18)	148

Symmetry codes: (i) 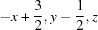; (ii) 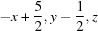; (iii) 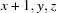; (iv) 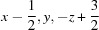.

**Fig. 1.**
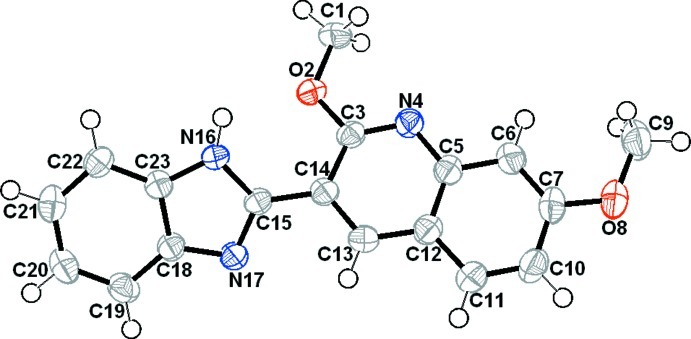
The structure of the title compound with displacement ellipsoids drawn at the 50% probability level.

**Fig. 2.**
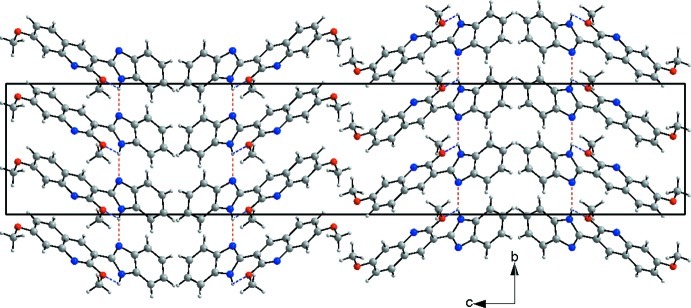
A diagram of the layered crystal packing of (I) viewed down the a axis and showing hydrogen bond [N—H···N and N—H···O] as dashed line.

## supplementary crystallographic information

### Comment

In the course of our program related to the synthesis of new suitably functionalized heterocyclic compounds of potential biological activity, (Benzerka *et al.*, 2012; Hayour *et al.*, 2011), we now report herein the synthesis and structure determination of the title compound, C_18_H_15_N_3_O_2_. The reactivity of this compound and its analogues toward nucleophiles is under investigation.

The molecular geometry and the atom-numbering scheme of (I) are shown in Fig. 1. In the asymmetric unit of title compound the dimethoxyquinoline unit bearing an benzo imidazol moiety. The two rings of quinolyl moiety are fused in an axial fashion and form a dihedral angle of 2.68 (4)°. The heterocycle ring of quinolyl unit form also with imidazol plane a dihedral angle of 24.09 (5)°. The crystal packing can be described as layers in zig zag parallel to (010) plane, along the *c* axis (Fig. 2). It is stabilized by intra and intermolecular hydrogen bond (N—H···N and N—H···O) and C—H···π stacking, resulting in the formation of infinite three-dimensional network linked these layers toghter and reinforcing a cohesion of structure. Hydrogen-bonding parameters are listed in table 1.

### Experimental

In first, malononitrile (1.0 mmol) was condensed with 2,7-dimethoxyquinolin-3-carbaldehyde (1 mmol) to give the corresponding Knoevenagel product in 97% yield. The oxidation of this one, under mild conditions, with 2.5 eq. of m.CPBA proceeded cleany, to afford corresponding 2,2-dicyano-3-(2,7-dimethoxyquinolin-3-yl)oxirane in 64% yield, according to the method reported by Fioraventi *et al.* (2006) In the next step, a mixture of 1.0 mmol. of 3-(2,7-dimethoxyquinolin-3-yl)oxirane-2,2-dicarbonitrile and 1.0 eq. of *o*-phenylenediamine dissolved in 30 ml of anhydrous acetonitrile was refluxed during 20 h. The title compound was successfully isolated by silica gel column chromatography using n.hexane/EtOAc (3:2) mixture as eluent in good yield (62%). Colourless blocks were obtained by crystallization (slow evaporation at room temperature) from a dichloromethane/methanol solution.

### Refinement

All non-H atoms were refined with anisotropic atomic displacement parameters. All H atoms were localized on Fourier maps but introduced in calculated positions and treated as riding on their parent C or N atom. (with C—H = 0.95 and 0.98 Å, N—H = 0.88 Å and *U*_iso_(H) =1.5 or 1.2(carrier atom)).

### Crystal data

**Table:**

C_18_H_15_N_3_O_2_	*F*(000) = 1280
*M**_r_* = 305.33	*D*_x_ = 1.306 Mg m^−^^3^
Orthorhombic, *P**b**c**a*	Mo *K*α radiation, λ = 0.71073 Å
Hall symbol: -P 2ac 2ab	Cell parameters from 3402 reflections
*a* = 6.7094 (2) Å	θ = 3.2–26.7°
*b* = 9.4134 (3) Å	µ = 0.09 mm^−^^1^
*c* = 49.1620 (16) Å	*T* = 150 K
*V* = 3104.99 (17) Å^3^	Block, colourless
*Z* = 8	0.51 × 0.29 × 0.09 mm

### Data collection

**Table:**

Bruker APEXII diffractometer	2696 reflections with *I* > 2σ(*I*)
Graphite monochromator	*R*_int_ = 0.035
CCD rotation images, thin slices scans	θ_max_ = 27.1°, θ_min_ = 3.2°
Absorption correction: multi-scan (*SADABS*; Sheldrick, 2002)	*h* = −8→8
*T*_min_ = 0.900, *T*_max_ = 0.992	*k* = −12→11
14322 measured reflections	*l* = −51→62
3398 independent reflections	

### Refinement

**Table:**

Refinement on *F*^2^	Primary atom site location: structure-invariant direct methods
Least-squares matrix: full	Secondary atom site location: difference Fourier map
*R*[*F*^2^ > 2σ(*F*^2^)] = 0.046	Hydrogen site location: inferred from neighbouring sites
*wR*(*F*^2^) = 0.112	H-atom parameters constrained
*S* = 1.03	*w* = 1/[σ^2^(*F*_o_^2^) + (0.0451*P*)^2^ + 1.4136*P*] where *P* = (*F*_o_^2^ + 2*F*_c_^2^)/3
3398 reflections	(Δ/σ)_max_ = 0.001
210 parameters	Δρ_max_ = 0.23 e Å^−^^3^
0 restraints	Δρ_min_ = −0.21 e Å^−^^3^

### Special details

**Table:**

Geometry. All e.s.d.'s (except the e.s.d. in the dihedral angle between two l.s. planes) are estimated using the full covariance matrix. The cell e.s.d.'s are taken into account individually in the estimation of e.s.d.'s in distances, angles and torsion angles; correlations between e.s.d.'s in cell parameters are only used when they are defined by crystal symmetry. An approximate (isotropic) treatment of cell e.s.d.'s is used for estimating e.s.d.'s involving l.s. planes.
Refinement. Refinement of *F*^2^ against ALL reflections. The weighted *R*-factor *wR* and goodness of fit *S* are based on *F*^2^, conventional *R*-factors *R* are based on *F*, with *F* set to zero for negative *F*^2^. The threshold expression of *F*^2^ > σ(*F*^2^) is used only for calculating *R*-factors(gt) *etc*. and is not relevant to the choice of reflections for refinement. *R*-factors based on *F*^2^ are statistically about twice as large as those based on *F*, and *R*- factors based on ALL data will be even larger.

### Fractional atomic coordinates and isotropic or equivalent isotropic displacement parameters (Å^2^)

**Table:**

	*x*	*y*	*z*	*U*_iso_*/*U*_eq_	
C1	1.2836 (2)	0.46202 (18)	0.63727 (4)	0.0326 (4)	
H1A	1.272	0.4041	0.6208	0.049*	
H1B	1.3178	0.4009	0.6527	0.049*	
H1C	1.3884	0.5334	0.6347	0.049*	
C3	1.0281 (2)	0.62110 (15)	0.62327 (3)	0.0232 (3)	
C5	1.0569 (2)	0.73868 (16)	0.58254 (3)	0.0258 (3)	
C6	1.1711 (3)	0.76468 (17)	0.55888 (3)	0.0301 (4)	
H6	1.2896	0.7122	0.5555	0.036*	
C7	1.1086 (3)	0.86695 (19)	0.54070 (3)	0.0329 (4)	
C9	1.3846 (3)	0.8225 (2)	0.51100 (4)	0.0453 (5)	
H9A	1.4838	0.8368	0.5254	0.068*	
H9B	1.4391	0.855	0.4936	0.068*	
H9C	1.3514	0.7213	0.5098	0.068*	
C10	0.9307 (3)	0.94447 (19)	0.54508 (3)	0.0357 (4)	
H10	0.8893	1.0141	0.5323	0.043*	
C11	0.8182 (3)	0.91890 (18)	0.56783 (3)	0.0335 (4)	
H11	0.6987	0.9709	0.5707	0.04*	
C12	0.8784 (2)	0.81534 (16)	0.58716 (3)	0.0270 (3)	
C13	0.7714 (2)	0.78642 (16)	0.61140 (3)	0.0264 (3)	
H13	0.6494	0.8344	0.6149	0.032*	
C14	0.8429 (2)	0.68943 (15)	0.62988 (3)	0.0231 (3)	
C15	0.7363 (2)	0.66229 (15)	0.65547 (3)	0.0219 (3)	
C18	0.5356 (2)	0.68915 (15)	0.68937 (3)	0.0239 (3)	
C19	0.3995 (3)	0.73837 (17)	0.70884 (3)	0.0310 (4)	
H19	0.3424	0.8305	0.7075	0.037*	
C20	0.3513 (3)	0.64821 (18)	0.73003 (3)	0.0327 (4)	
H20	0.259	0.6788	0.7435	0.039*	
C21	0.4363 (3)	0.51183 (18)	0.73215 (3)	0.0307 (4)	
H21	0.3998	0.4528	0.747	0.037*	
C22	0.5711 (2)	0.46135 (16)	0.71324 (3)	0.0266 (3)	
H22	0.6275	0.3691	0.7147	0.032*	
C23	0.6202 (2)	0.55309 (15)	0.69194 (3)	0.0225 (3)	
N4	1.12891 (19)	0.64018 (13)	0.60072 (3)	0.0254 (3)	
N16	0.74790 (19)	0.53920 (13)	0.67005 (2)	0.0229 (3)	
H16	0.8226	0.465	0.6662	0.027*	
N17	0.6102 (2)	0.75567 (13)	0.66624 (3)	0.0253 (3)	
O2	1.09768 (16)	0.53168 (11)	0.64249 (2)	0.0285 (3)	
O8	1.2084 (2)	0.90194 (14)	0.51729 (2)	0.0424 (3)	

### Atomic displacement parameters (Å^2^)

**Table:**

	*U*^11^	*U*^22^	*U*^33^	*U*^12^	*U*^13^	*U*^23^
C1	0.0285 (9)	0.0335 (9)	0.0357 (9)	0.0116 (7)	0.0000 (7)	0.0024 (7)
C3	0.0269 (8)	0.0181 (7)	0.0247 (8)	−0.0009 (6)	−0.0013 (7)	−0.0007 (6)
C5	0.0301 (8)	0.0239 (7)	0.0234 (8)	−0.0010 (6)	−0.0007 (7)	−0.0015 (6)
C6	0.0320 (8)	0.0325 (9)	0.0257 (8)	0.0016 (7)	0.0022 (7)	−0.0016 (7)
C7	0.0396 (10)	0.0378 (9)	0.0213 (8)	−0.0032 (8)	0.0024 (7)	0.0019 (7)
C9	0.0401 (10)	0.0612 (13)	0.0344 (10)	0.0042 (9)	0.0114 (9)	0.0084 (9)
C10	0.0455 (10)	0.0363 (9)	0.0254 (9)	0.0032 (8)	−0.0019 (8)	0.0065 (7)
C11	0.0373 (9)	0.0348 (9)	0.0285 (9)	0.0071 (8)	−0.0002 (8)	0.0043 (7)
C12	0.0322 (8)	0.0244 (8)	0.0245 (8)	−0.0002 (7)	−0.0010 (7)	0.0001 (6)
C13	0.0277 (8)	0.0229 (7)	0.0285 (8)	0.0029 (6)	0.0007 (7)	−0.0006 (6)
C14	0.0250 (8)	0.0178 (7)	0.0265 (8)	−0.0023 (6)	0.0016 (7)	−0.0019 (6)
C15	0.0240 (7)	0.0170 (7)	0.0247 (7)	−0.0018 (6)	−0.0006 (6)	−0.0001 (6)
C18	0.0260 (8)	0.0195 (7)	0.0261 (8)	−0.0023 (6)	0.0019 (7)	−0.0006 (6)
C19	0.0319 (9)	0.0262 (8)	0.0350 (9)	0.0014 (7)	0.0063 (8)	−0.0037 (7)
C20	0.0313 (9)	0.0364 (9)	0.0303 (9)	−0.0031 (7)	0.0085 (7)	−0.0052 (7)
C21	0.0347 (9)	0.0325 (8)	0.0250 (8)	−0.0091 (7)	0.0020 (7)	0.0002 (7)
C22	0.0297 (8)	0.0233 (7)	0.0269 (8)	−0.0029 (6)	−0.0022 (7)	0.0015 (6)
C23	0.0232 (7)	0.0204 (7)	0.0239 (8)	−0.0036 (6)	−0.0002 (6)	−0.0024 (6)
N4	0.0271 (7)	0.0239 (6)	0.0251 (7)	−0.0003 (5)	0.0009 (6)	0.0005 (5)
N16	0.0256 (6)	0.0184 (6)	0.0246 (7)	0.0026 (5)	0.0018 (6)	0.0011 (5)
N17	0.0273 (7)	0.0195 (6)	0.0291 (7)	−0.0002 (5)	0.0049 (6)	0.0009 (5)
O2	0.0280 (6)	0.0260 (6)	0.0317 (6)	0.0061 (5)	0.0031 (5)	0.0067 (5)
O8	0.0475 (8)	0.0515 (8)	0.0282 (7)	0.0046 (6)	0.0086 (6)	0.0112 (6)

### Geometric parameters (Å, º)

**Table:**

C1—O2	1.4327 (19)	C11—H11	0.95
C1—H1A	0.98	C12—C13	1.418 (2)
C1—H1B	0.98	C13—C14	1.375 (2)
C1—H1C	0.98	C13—H13	0.95
C3—N4	1.311 (2)	C14—C15	1.470 (2)
C3—O2	1.3490 (18)	C15—N17	1.3297 (19)
C3—C14	1.436 (2)	C15—N16	1.3646 (18)
C5—N4	1.376 (2)	C18—N17	1.3912 (19)
C5—C6	1.414 (2)	C18—C19	1.402 (2)
C5—C12	1.416 (2)	C18—C23	1.407 (2)
C6—C7	1.379 (2)	C19—C20	1.382 (2)
C6—H6	0.95	C19—H19	0.95
C7—O8	1.372 (2)	C20—C21	1.409 (2)
C7—C10	1.416 (3)	C20—H20	0.95
C9—O8	1.433 (2)	C21—C22	1.381 (2)
C9—H9A	0.98	C21—H21	0.95
C9—H9B	0.98	C22—C23	1.397 (2)
C9—H9C	0.98	C22—H22	0.95
C10—C11	1.371 (2)	C23—N16	1.3817 (19)
C10—H10	0.95	N16—H16	0.88
C11—C12	1.420 (2)		
			
O2—C1—H1A	109.5	C14—C13—H13	119.8
O2—C1—H1B	109.5	C12—C13—H13	119.8
H1A—C1—H1B	109.5	C13—C14—C3	116.74 (14)
O2—C1—H1C	109.5	C13—C14—C15	120.74 (14)
H1A—C1—H1C	109.5	C3—C14—C15	122.49 (13)
H1B—C1—H1C	109.5	N17—C15—N16	112.88 (13)
N4—C3—O2	119.97 (14)	N17—C15—C14	122.37 (13)
N4—C3—C14	125.19 (14)	N16—C15—C14	124.70 (13)
O2—C3—C14	114.84 (13)	N17—C18—C19	130.07 (14)
N4—C5—C6	117.44 (14)	N17—C18—C23	109.77 (13)
N4—C5—C12	122.40 (14)	C19—C18—C23	120.15 (14)
C6—C5—C12	120.13 (14)	C20—C19—C18	117.66 (15)
C7—C6—C5	119.29 (16)	C20—C19—H19	121.2
C7—C6—H6	120.4	C18—C19—H19	121.2
C5—C6—H6	120.4	C19—C20—C21	121.37 (16)
O8—C7—C6	124.28 (16)	C19—C20—H20	119.3
O8—C7—C10	114.55 (15)	C21—C20—H20	119.3
C6—C7—C10	121.17 (16)	C22—C21—C20	121.94 (15)
O8—C9—H9A	109.5	C22—C21—H21	119
O8—C9—H9B	109.5	C20—C21—H21	119
H9A—C9—H9B	109.5	C21—C22—C23	116.50 (15)
O8—C9—H9C	109.5	C21—C22—H22	121.7
H9A—C9—H9C	109.5	C23—C22—H22	121.7
H9B—C9—H9C	109.5	N16—C23—C22	132.20 (14)
C11—C10—C7	119.87 (16)	N16—C23—C18	105.44 (13)
C11—C10—H10	120.1	C22—C23—C18	122.36 (14)
C7—C10—H10	120.1	C3—N4—C5	117.44 (13)
C10—C11—C12	120.65 (16)	C15—N16—C23	107.05 (12)
C10—C11—H11	119.7	C15—N16—H16	126.5
C12—C11—H11	119.7	C23—N16—H16	126.5
C5—C12—C13	117.74 (14)	C15—N17—C18	104.86 (12)
C5—C12—C11	118.88 (15)	C3—O2—C1	117.47 (12)
C13—C12—C11	123.36 (15)	C7—O8—C9	117.27 (14)
C14—C13—C12	120.40 (14)		
			
N4—C5—C6—C7	−177.24 (15)	C23—C18—C19—C20	−0.8 (2)
C12—C5—C6—C7	1.1 (2)	C18—C19—C20—C21	0.3 (3)
C5—C6—C7—O8	179.35 (15)	C19—C20—C21—C22	−0.1 (3)
C5—C6—C7—C10	−1.1 (3)	C20—C21—C22—C23	0.4 (2)
O8—C7—C10—C11	−179.94 (16)	C21—C22—C23—N16	179.34 (16)
C6—C7—C10—C11	0.5 (3)	C21—C22—C23—C18	−1.0 (2)
C7—C10—C11—C12	0.1 (3)	N17—C18—C23—N16	0.35 (17)
N4—C5—C12—C13	−0.8 (2)	C19—C18—C23—N16	−179.03 (14)
C6—C5—C12—C13	−179.12 (14)	N17—C18—C23—C22	−179.41 (14)
N4—C5—C12—C11	177.74 (15)	C19—C18—C23—C22	1.2 (2)
C6—C5—C12—C11	−0.6 (2)	O2—C3—N4—C5	−176.33 (13)
C10—C11—C12—C5	−0.1 (3)	C14—C3—N4—C5	3.4 (2)
C10—C11—C12—C13	178.40 (16)	C6—C5—N4—C3	176.76 (14)
C5—C12—C13—C14	1.5 (2)	C12—C5—N4—C3	−1.6 (2)
C11—C12—C13—C14	−176.94 (15)	N17—C15—N16—C23	−0.02 (17)
C12—C13—C14—C3	0.0 (2)	C14—C15—N16—C23	−177.42 (14)
C12—C13—C14—C15	177.97 (14)	C22—C23—N16—C15	179.52 (16)
N4—C3—C14—C13	−2.7 (2)	C18—C23—N16—C15	−0.20 (16)
O2—C3—C14—C13	177.08 (13)	N16—C15—N17—C18	0.23 (17)
N4—C3—C14—C15	179.41 (14)	C14—C15—N17—C18	177.70 (14)
O2—C3—C14—C15	−0.8 (2)	C19—C18—N17—C15	178.94 (17)
C13—C14—C15—N17	−22.2 (2)	C23—C18—N17—C15	−0.36 (17)
C3—C14—C15—N17	155.59 (15)	N4—C3—O2—C1	0.8 (2)
C13—C14—C15—N16	154.92 (15)	C14—C3—O2—C1	−179.00 (13)
C3—C14—C15—N16	−27.2 (2)	C6—C7—O8—C9	2.5 (3)
N17—C18—C19—C20	179.94 (16)	C10—C7—O8—C9	−177.05 (16)

### Hydrogen-bond geometry (Å, º)

Cg1, Cg2 and Cg4 are the centroids of the N16/N17/C15/C18/C23, N4/C3/C5/C12–C14 and C18–C23 rings, respectively.

**Table:**

*D*—H···*A*	*D*—H	H···*A*	*D*···*A*	*D*—H···*A*
N16—H16···N17^i^	0.88	2.02	2.8397 (17)	154
N16—H16···O2	0.88	2.27	2.7107 (17)	111
C1—H1*A*···*Cg*2^ii^	0.98	2.67	3.3101 (18)	123
C1—H1*C*···*Cg*1^iii^	0.98	2.82	3.4955 (17)	127
C20—H20···*Cg*4^iv^	0.95	2.99	3.8271 (18)	148

Symmetry codes: (i) −*x*+3/2, *y*−1/2, *z*; (ii) −*x*+5/2, *y*−1/2, *z*; (iii) *x*+1, *y*, *z*; (iv) *x*−1/2, *y*, −*z*+3/2.
